# Associations between disordered eating behaviour and sexual behaviour amongst emerging adults attending a tertiary education institution in Coastal Kenya

**DOI:** 10.1371/journal.pone.0301436

**Published:** 2024-06-11

**Authors:** Stevenson K. Chea, Adama Kazienga, Eunice A. Oyugi, Isaac Menza, Carophine Nasambu, Fauz Ibrahim, Osman A. Abdullahi, Amin S. Hassan, Amina Abubakar, Kristien Michielsen, Souheila Abbeddou

**Affiliations:** 1 Department of Nursing Sciences, School of Health and Human Sciences, Pwani University, Kilifi, Kenya; 2 Department of Public Health and Primary Care, Faculty of Medicine and Health Sciences, Ghent University, Ghent, Belgium; 3 Department of Translational Physiology, Infectiology and Public Health, Ghent University, Merelbeke, Belgium; 4 Centre for Geographic Medicine Research, Kenya Medical Research Institute, Kilifi, Kenya; 5 County Department of Health, Kilifi, Kenya; 6 Department of Public Health, School of Health and Human Sciences, Pwani University, Kilifi, Kenya; 7 Department of Psychiatry, University of Oxford, Oxford, United Kingdom; 8 Institute for Human Development, Aga Khan University, Nairobi, Kenya; 9 Department of Neurosciences, Institute for Family and Sexuality Studies, Faculty of Medicine, Catholic University of Leuven (KU Leven), Leven, Belgium; University of Ghana, GHANA

## Abstract

**Background:**

Sexual behavior (SB) is a well-documented pathway to HIV acquisition in emerging adults and remains common amongst African emerging adults. Previous research in high-income countries indicates a correlation between disordered eating behavior (DEB) and engaging in sexual behaviors. We aimed to describe the relationship between DEB and SB amongst emerging adults attending a tertiary educational institution at the Kenyan Coast.

**Methods:**

We applied a cross-sectional design nested in a young adults’ cohort study. Eligibility included sexually active emerging adults aged 18–24 years. Three DEBs (emotional, restrained and external eating) were assessed using the Dutch Eating Behavior Questionnaire and analysed using exploratory factor analysis. Seven SB indicators were assessed: non-condom use, casual sex, multiple sex partners, transactional sex, group sex, age-disparate relationship and anal sex, and grouped into low vs. high SB using latent class analysis. Logistic regression was used to assess the association between DEB and SB.

**Results:**

Of 273 eligible participants (female, n = 110 [40.3%]), the mean of emotional, restrained and external eating was 1.9 [0.6], 2.0 [0.6] and 3.0 [0.5] respectively. Overall, 57 (20.9%) were grouped into the latent high SB class. Emotional (Adjusted odds ratio, AOR [95% confidence interval, CI]: 1.0 [0.9–1.0], p = 0.398), restrained (AOR, 1.0 [CI: 0.9–1.1], p = 0.301) and External (AOR, 1.0 [CI: 0.8–1.2], p = 0.523) eating were not independently associated with latent high SB.

**Conclusion:**

There was no significant association between DEB and SB in this study sample. In low- and middle-income countries like Kenya, interventions targeted at DEB among emerging adults towards controlling SB are unnecessary.

## Background

Sexual behavior (SB) is a well-documented pathway to HIV acquisition in emerging adults [1–7]. Evidence suggests that SB is common amongst emerging adults in sub Saharan Africa (sSA) [8] with non-condom use ranging from 4.0% in South Africa [9] to 94.5% in Madagascar [10]. It is not surprising that new HIV infections amongst young people including emerging adults contributed 31% of new HIV infections in sSA in 2019 [11]. Due to their developing brains [12, 13], emerging adults have high propensity to impulsivity which increases the risk of SB [12–14]. Further, emerging adults in tertiary institutions of learning enjoy freedom from close parental monitoring which may encourage SB [15].

SB in emerging adults in sSA has been well studied. Previous studies have explored several underlying risks including socio-demographic factors like age [7, 16], education level [17, 18] and socio-economic status [7, 19] as well as relationship/behavioral factors like alcohol/substance abuse [16, 17], sexual violence [20, 21] and number of sexual partners [22]. Others are knowledge/attitude/belief factors like knowledge of HIV status [23], HIV knowledge [24] and perceived HIV risk [24]; family/community factors like social network affiliation [25–27], and mental/physical health factors like sexually transmitted infections (STIs) [28], post-traumatic stress disorder [29] and depression [16, 29].

Neglected, yet important especially in sSA where there is increased urbanization, which is associated with lifestyle changes, is disordered eating behavior (DEB). DEB like emotional eating, restrained eating and external eating have been associated with SB in high income countries [30]. For example, Fergus (2019) found that young women with eating disorders were more likely to engage in sexual behaviors, such as having multiple sexual partners and not using condoms[31].

Further, previous studies have shown that DEB is prevalent among African emerging adults ranging from 16% in a Nigerian study that enrolled undergraduate students [32] to 63% among South African female university students [33]. With the high SB reported in African emerging adults [8], it remains unclear if an association between DEB and SB exists. We aimed to describe the relationship between three DEB constructs (emotional, restrictive and external eating) and SB amongst emerging adults attending a tertiary education institution in Coastal Kenya.

## Methods

### Study design

We applied a cross sectional study design nested within a young adults’ cohort study (YACOS). In brief, YACOS is a longitudinal study that aimed to characterize sexually transmitted infections (STIs) amongst emerging adults at Pwani University, a tertiary learning institution located in Kilifi County, Coastal Kenya. The University has an estimated population of 8,000 students [34] and enrols an estimated 2000 students every academic year. Pwani University students aged 18–24 years and willing to undergo STI/HIV screening were eligible. Students who reported to know their HIV-1 positive infection status were excluded since they already had the primary end point (HIV). Eligible volunteers were enrolled and followed up three-monthly. For the nested sub-study, eligibility included sexually active participants (had sexual intercourse in the last 3 months [n = 273]). Given that the student population is largely homogenous, recruitment was done consecutively between July 29^th^ 2021 and June 22^nd^ 2022.

### Study procedures

To popularize the study, study fliers were randomly distributed to students and others posted on student notice boards. Additionally, soft copies of the fliers were posted in student whatsapp groups. During recruitment, the study counsellor gave a brief overview of the study and subsequently screened them for eligibility. Next, eligible students were taken through the written informed consent process. Consenting students had their anthropometric indicators (weight, height, and waist and hip circumferences) assessed by the study counsellor. The study counsellor then handed over participants to the study clinician for the second set of assessments which included, first, a repeat of the anthropometrics for validation, eating behaviour, mental health, SB, substance use, and clinical assessments. Mental health, substance use and SB assessments were conducted through an audio-computer assisted self-interview (ACASI) [35]. The study clinician transferred participants to the study counsellor for HIV self-testing. The study counsellor provided pretest counselling, demonstrated self-test use, and captured results. Post-test counselling was then offered. All HIV tests were done using the OraQuick® Rapid HIV 1/2 antibody test as per national guidelines [36].

### Ethical considerations

Ethical approval was obtained from the KEMRI Scientific and Ethics Research Unit reference number KEMRI/SERU/CGMR-C/166/3925. Participants reporting STI symptoms were treated as per national guidelines [37]. Participants who turned out to be HIV-infected were referred to the Pwani University HIV voluntary counselling and testing centre for a confirmatory test in line with Kenya national guidelines [36]. Each participant received a reimbursement to compensate for their time. Investigators had no access to information that could identify individual participants during or after data collection. All participants gave a written informed consent to participate in the study.

### Tools of data collection

#### Sexual behavior (SB)

SB was assessed using a customized hybrid of the Tambua Mapema Plus (TMP) trial Risk assessment tool [38] and the Kilifi Health Risk Behavior Questionnaire (KRIBE-Q) [39]. In brief, the TMP tool focuses on sexual risk assessment. The KRIBE-Q has previously been validated and adopted for use amongst adolescents and young adults in Kilifi and proved to be a reliable tool (Gwet ACI = 0.82) [39]. Seven SB outcome indicators were assessed; i) non-condom use (defined as not using a condom at last sex in the last 3 months, ii) casual sex (defined as sex with a short term partner in the past 3 months, where short term means a partner one has sex with for the first time and they do not intend to have any further sexual contact with them), iii) multiple sex partners (defined as having had more than one sex partner in the last 3 months), iv) transactional sex (defined as having been paid and/or paid someone money, bought gifts, bought alcohol or supported their living expenses for sex in the last 3 months), v) group sex (having had sex with more than one person at the same time in past 3 months), vi) age-disparate relationship (having a sex partner who is more than 10 years older in past 3 months), and vii) anal sex (defined as having had anal sex in past 3 months). Although sex can be relatively safe even with most of the SBs (except non-condom use), we considered all the seven behaviours to be risky like it has been done in previous studies [21, 40–45].

#### Disordered eating behaviour (DEB)

Emotional, restrictive and external eating were measured using the Dutch eating behaviour scale (DEBQ) [46]. The DEBQ was designed to measure eating patterns that may contribute to the development of overweight and obesity. In brief, the DEBQ is a 33-item, self-assessment scale for assessing three eating behavior domains: the restrained subscale (10 items), the emotional eating subscale (13 items), and the external eating subscale (10 items). The emotional eating pattern corresponds to the tendency towards overeating in response to negative emotions. Restrained eating refers to restriction of food intake in order to prevent weight gain. External eating means eating more in response to external food cues such as food’s sight, smell and taste. Participants were required to rate each item on a 5-point Likert scale ranging from 1 (seldom/never) to 5 (very often). Item scores for each subscale were added to obtain an overall subscale score that was then divided by the number of subscale items to calculate score per subscale. Higher scores indicate a greater tendency to exhibit the subscale behavior. Binge eating, defined as eating an amount of food that others would regard as unusually large, was assessed by an additional question on the DEBQ scale.

#### Anthropometric indicators

Anthropometrics were measured in line with the World Health Organization (WHO) protocols [47]. All measurements were independently taken by two assessors to the nearest 0.1 unit. Weight was measured using a digital scale (Model 769, Seca, Hamburg, Germany). Height was measured using a portable adult height scale (Model 37–113, HEMC, Noida, India). Waist and hip circumference were measured using a measuring tape. The mean of the two sets of measurements from the two assessors was used to compute Body Mass Index (BMI) [defined as weight in kilograms divided by the square of height in meters]) and Waist-Hip-Ratio (WHR). A BMI of <18.5 kg/m^2^, 18.5–25 kg/m^2^ and >25 kg/m^2^ was categorized as underweight, healthy, and overweight respectively [48]. A WHR of <0.95 or <0.8 for males and females respectively was classified as low risk for cardiovascular complications [47].

#### Mental health disorders

Binge drinking, and substance use were assessed. Binge drinking, defined as having six or more drinks on one occasion, was assessed using the Alcohol Use Disorders Identification Test (AUDIT) [49]. The screening tool was designed to be used globally and has been proven to be reliable and valid in detecting alcoholic tendencies [49–51]. Each AUDIT response is scored based on a point scale from 0 to 4. A score of 0, 1–7, 8–14 and above 14 represents abstainer, low risk consumption, harmful consumption and alcohol dependence respectively [49].

#### Other measures

These were administered through interviews and ACASI and included self-reported STI symptoms, socio-demographic characteristics, HIV risk perception, gambling and injuries. Based on the Kenyan national STI guidelines, the presence of at least one of the following 10 symptoms in the last three months was regarded as having STI: urethral discharge, genital sores, dysuria (pain while urinating), testicular pain/tenderness, vaginal itching/burning, vaginal discharge, lower abdominal pain, pain while having sex, rectal discharge/bleeding and peri-anal sores/growths [37, 52].

### Analysis

In a first step, we ran a latent class analysis (LCA) [53] to identify SB latent classes based on the seven SB indicators. Secondly, we ran an exploratory factor analysis [54] to test the factorial structure of the DEBQ items. Based on exploratory factor analysis, the 33 DEBQ items produced 9 factors with eigen values of >1.0. Out of all 33 items, 15 were not included in the final model due to factor loadings of <0.50. Six factors were dropped due to low composite reliability. The remaining 3 factors (factor 1–3) each had a composite reliability of >0.70 and were therefore included in the final model (Table 1). In factor 1, 11 items had loadings of ≥0.50. In factor 2 and 3, there were 5 and 2 items respectively (Table 1). All DEBQ items loaded into the 3 factors the same way as in the original DEBQ tool hence the names of factors generated remained unchanged (i.e. Factor 1 [Emotional eating; 11 items]; Factor 2 [Restrained eating; 5 items]; Factor 3 [External eating; 2 items]). Next, we ran a confirmatory factor analysis [55] to assess the fit of the factorial structure identified in the exploratory factor analysis. Good model fit parameters were i) a Tucker-Lewis index ≥0.95, ii) comparative fit index ≥0.95, iii) root mean square error of approximation <0.06 and iv) the smallest root mean square residual possible. The average variance extracted (AVE) which should be ≥0.5 for convergent validity to be assured was 0.671 for external eating but low for emotional eating and restrained eating (0.401 and 0.462 respectively). However, with AVE values ≥0.4 and CR ≥0.6 (0.8770 and 0.8002 for emotional eating and restrained eating respectively), convergent validity was still present [56]. The final 18 –item model demonstrated acceptable parameters (Table 2 and S1 and S2 Tables).

**Table 1:** Loadings for all DEBQ items in the 9 factors retained after exploratory factor analysis (n = 273).

DEBQ Items^a^	Factor 1	Factor 2	Factor 3	Factor 4	Factor 5	Factor 6	Factor 7	Factor 8	Factor 9
Item 1	0.638*	- 0.016	0.122	- 0.142	- 0.023	- 0.093	- 0.004	0.138	-0.139
Item 2	0.038	0.021	0.862*	0.013	0.001	- 0.011	- 0.057	- 0.067	0.082
Item 3	0.259	0.061	0.133	0.083	0.606*	- 0.324	0.163	- 0.085	- 0.162
Item 4	- 0.041	0.384	0.094	0.123	- 0.095	0.545*	- 0.224	- 0.154	- 0.099
Item 5	0.642*	0.083	0.259	- 0.045	- 0.156	- 0.067	0.177	- 0.079	0.059
Item 6	0.059	- 0.076	0.736*	0.173	0.245	0.196	0.022	0.054	0.002
Item 7	0.083	0.664*	- 0.023	- 0.071	- 0.187	0.087	0.028	0.037	- 0.026
Item 8	0.508*	- 0.022	0.147	- 0.043	0.143	0.154	0.229	- 0.034	0.142
Item 9	0.024	- 0.103	0.477	0.433	0.081	- 0.146	0.200	0.224	0.145
Item 10	0.687*	- 0.134	- 0.025	- 0.007	0.100	0.214	- 0.021	0.121	0.208
Item 11	0.035	0.351	0.007	- 0.024	0.526*	0.077	- 0.286	0.129	0.147
Item 12	0.001	- 0.022	0.388	0.317	- 0.076	- 0.052	0.273	0.357	- 0.106
Item 13	0.709*	0.072	0.070	0.025	0.142	- 0.111	0.056	0.088	0.181
Item 14	0.041	0.167	0.004	- 0.089	- 0.025	0.001	- 0.028	0.824*	- 0.035
Item 15	- 0.098	- 0.037	0.068	0.717*	0.052	0.161	0.044	- 0.021	- 0.009
Item 16	0.338	- 0.042	0.109	0.076	0.070	0.690*	0.219	0.067	- 0.039
Item 17	0.047	0.399	- 0.107	- 0.027	0.164	0.114	0.583*	- 0.042	0.020
Item 18	0.110	- 0.066	0.409	0.394	0.227	0.051	- 0.172	- 0.094	- 0.370
Item 19	0.092	0.362	0.058	- 0.129	0.214	0.294	0.022	- 0.105	0.329
Item 20	0.624*	0.029	0.101	- 0.026	0.049	0.155	0.346	- 0.003	- 0.255
Item 21	- 0.046	0.086	0.134	0.160	- 0.070	- 0.054	- 0.010	- 0.051	0.740*
Item 22	0.002	0.765*	0.061	- 0.084	- 0.124	- 0.035	0.017	0.045	0.077
Item 23	0.729*	0.009	- 0.079	0.068	0.172	0.185	0.061	0.025	0.081
Item 24	- 0.018	- 0.008	0.219	0.604*	0.137	0.220	- 0.093	- 0.306	- 0.036
Item 25	0.769*	0.041	0.097	- 0.064	- 0.018	- 0.037	- 0.104	- 0.058	- 0.049
Item 26	- 0.033	0.814*	- 0.006	- 0.012	0.099	0.030	0.085	0.068	0.057
Item 27	- 0.013	- 0.024	0.293	0.117	0.641*	0.231	0.166	- 0.064	- 0.058
Item 28	0.542*	- 0.006	0.007	0.126	0.068	0.049	0.542	- 0.035	0.046
Item 29	0.012	0.781*	- 0.058	0.033	0.186	- 0.056	0.065	0.019	- 0.055
Item 30	0.606*	- 0.012	- 0.071	0.009	0.116	0.152	- 0.118	0.063	- 0.072
Item 31	0.081	0.586*	- 0.160	0.080	0.026	0.106	- 0.182	0.385	0.088
Item 32	0.758*	0.052	- 0.129	0.061	- 0.117	0.023	- 0.001	- 0.044	- 0.260
Item 33	0.074	0.002	0.055	0.702*	- 0.029	- 0.136	0.037	0.017	0.205
Eigen value	5.54823	3.54233	3.05325	1.45832	1.29622	1.20231	1.15840	1.06005	1.00009

^a^Original DEBQ item numbers: Restrained eating [Item 4,7,11,14,17,19,22,26,29,31]; emotional eating [Item 1,3,5,8,10,13,16,20,23,25,28,30,32] external eating [Item 2,6,9,12,15, 18, 21, 24,27,33]

*Loadings >0.50

**Table 2:** Construct validity and reliability of the final DEBQ model (n = 273).

Factors		Parameters	
	AVE	Square root of AVE	CR
Emotional eating	0.401	0.6332	0.8770
Restrained eating	0.462	0.6797	0.8002
External eating	0.671	0.8191	0.7374

AVE–Average Variance Extracted CR–Composite Reliability

Convergent validity- Present if AVE is ≥0.5; 0.4 acceptable if CR is above 0.6

Discriminant validity (Square-root of AVE)- Present if correlation between latent constructs is less than square-root of AVE

Finally, we conducted logistic regression to assess the association between DEB (emotional, restrained and external eating) and SB controlling for socio-demographic characteristics, anthropometric indicators, mental health disorders, HIV risk perception, gambling, injuries and substance use. We used a two-step approach to select the exposures to be included in regression analysis. First, exposures considered clinically/physiologically plausible were selected for inclusion in the univariable model. Next, exposure variables with a p-value of <0.2 in univariable analysis were carried forward to the multivariable model. This approach ensured that only the “strong” variables were selected. We tested the collinearity between the variables controlled for in the final model. With the exception of sex and WHR which have a correlation of 0.6, the rest of the pairs have a correlation of below 0.5 suggesting that the coefficients obtained are stable. Analyses were done using Stata 15.0 (StataCorp.2017. Stata Statistical Software: Release 15. College Station, TX: StataCorp LLC. 2019). A correlation with p-value of less than 5% was considered statistically significant.

## Results

### Characteristics of study participants

**Fig 1:**
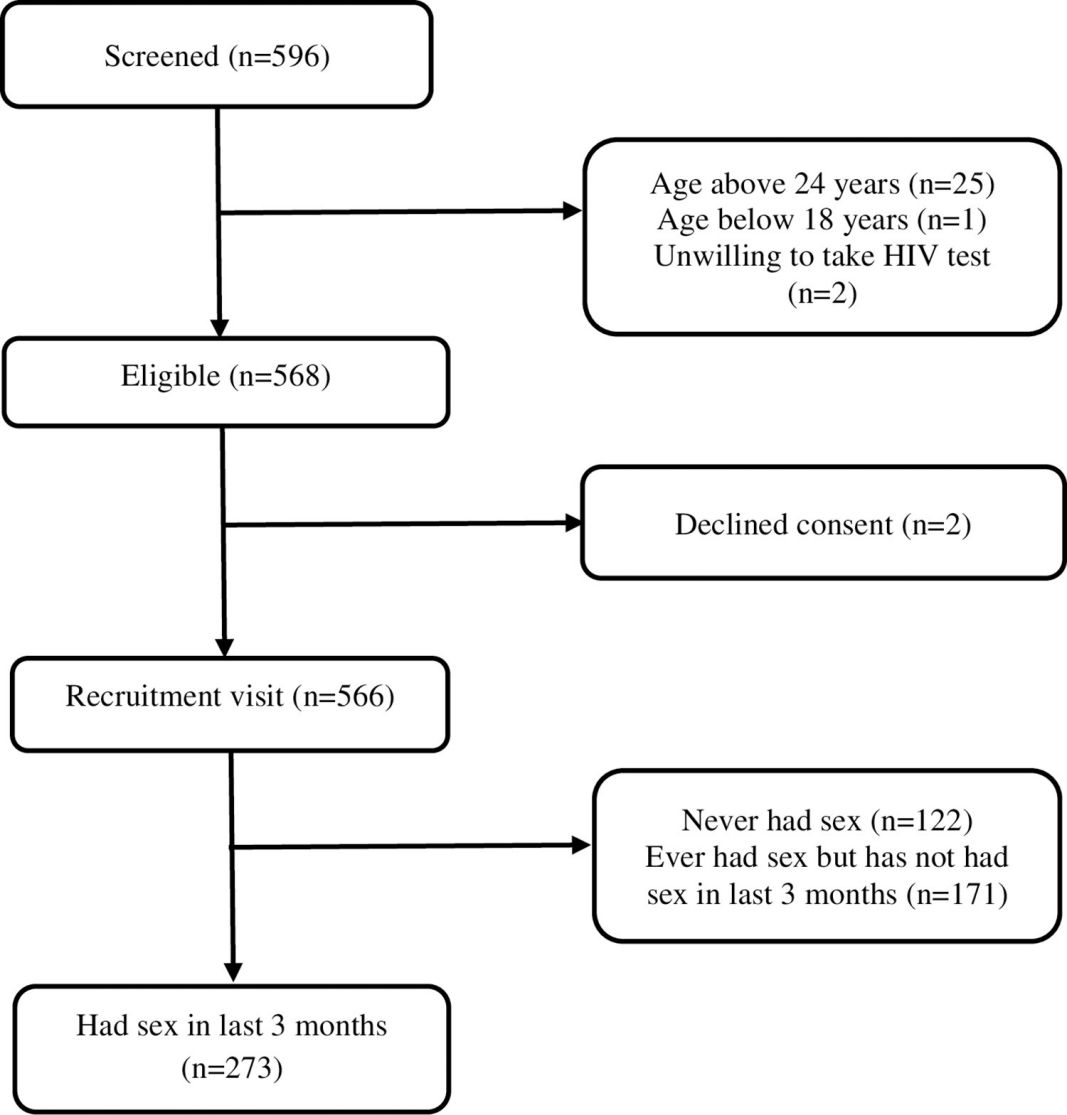
Flow diagram showing enrolment of young adults aged 18–24 years recruited from Pwani University.

Overall, 273 participants were eligible for the sub-study (Fig 1). The mean age, BMI and WHR was 21.0 [1.6] years, 21.5 [2.7] kg/m^2^ and 0.8 [0.0] respectively. Overall, majority of the participants were male (163 [59.7%]), aged 21–24 years old (160 [58.6%]), had never been tested for HIV infection (212 [77.7%]) and did not report any STI symptoms in the past 3 months (172 [63.0%]) (Table 3).

**Table 3:** Socio-demographic, clinical and psychosocial characteristics of sexually active emerging adults aged 18–24 years attending a tertiary learning institution in Coastal Kenya (N = 273).

Characteristics	Category	Male	Female	Overall
		(n = 163)	(n = 110)	(N = 273)
		N [%]	N [%]	N [%]
***Socio-demographic and clinical indicators***				
Age [years] Mean	-	21.2 [1.5]	20.7 [1.5]	21.0 [1.6]
Age group [Years]	18–20	57 [35.0]	56 [50.9]	113 [41.4]
	21–24	106 [65.0]	54 [49.1]	160 [58.6]
Year of study	Year 1 & 2	92 [56.4]	63 [57.3]	155 [56.8]
	Year 3 & 4	71 [43.6]	47 [42.7]	118 [43.2]
Ever been tested for HIV	No	126 [77.3]	86 [78.2]	212 [77.7]
	Yes	37 [22.7]	24 [21.8]	61 [22.3]
Have children?	No	156 [95.7]	99 [90.0]	255 [93.4]
	Yes	7 [4.3]	11 [10.0]	18 [6.6]
Parents alive?	Both parents alive	128 [78.5]	91 [82.7]	219 [80.2]
	One or both parents dead	35 [21.5]	19 [17.3]	54 [19.8]
Living arrangement	In campus	17 [10.4]	17 [15.4]	34 [12.5]
	Outside campus	146 [89.6]	93 [84.6]	239 [87.5]
Religion	Catholic	48 [29.5]	25 [22.8]	73 [26.7]
	Protestant or other Christian	113 [69.3]	81 [73.6]	194 [71.1]
	Muslim	2 [1.2]	4 [3.6]	6 [2.2]
STI symptoms past 3 months	No	138 [84.7]	34 [30.9]	172 [63.0]
	Yes	25 [15.3]	76 [69.1]	101 [37.0]
Perceived chances of getting HIV	Small chance	135 [82.8]	95 [86.4]	230 [84.3]
	Great chance	28 [17.2]	15 [13.6]	43 [15.7]
Ever taken PEP and/or PreP	No	155 [95.1]	108 [98.2]	263 [96.3]
	Yes	8 [4.9]	2 [1.8]	10 [3.7]
Ever taken part in gambling	No	39 [23.9]	91 [82.7]	130 [47.6]
	Yes	124 [76.1]	19 [17.3]	143 [52.4]
Seriously injured past 3 months	No	139 [85.3]	99 [90.0]	238 [87.2]
	Yes	24 [14.7]	11 [10.0]	35 [12.8]
Binge eating ever	No	88 [54.0]	67 [60.9]	155 [56.8]
	Yes	75 [46.0]	43 [39.1]	118 [43.2]
***Anthropometric indicators***				
Body Mass Index [kg/m^2^] Mean	-	21.1 [2.3]	22.1 [3.1]	21.5 [2.7]
Body Mass Index categories	Underweight [<18.5]	17 [10.4]	9 [8.2]	26 [9.5]
[kg/m^2^]	Healthy [18.5–25]	136 [83.4]	81 [73.6]	217 [79.5]
	Overweight [>25]	10 [6.1]	20 [18.2]	30 [11.0]
Waist-Hip Ratio (WHR, Mean/SD)	-	0.8 [0.0]	0.8 [0.0]	0.8 [0.0]
Waist-Hip Ratio categories	Low risk	163 [100.0]	53 [48.2]	216 [79.1]
	High risk	0 [0.0]	57 [51.8]	57 [20.9]
***Mental health indicators***				
Binge drinking past 3 months	No alcohol last 3 months	70 [42.9]	76 [69.0]	146 [53.5]
	No	66 [40.5]	28 [25.5]	94 [34.4]
	Yes	27 [16.6]	6 [5.5]	33 [12.1]
Marijuana use past 3 months	Never used marijuana	105 [64.4]	95 [86.4]	200 [73.3]
	No	18 [11.1]	3 [2.7]	21 [7.7]
	Yes	40 [24.5]	12 [10.9]	52 [19.0]
Tobacco use past 3 months	Never used tobacco	122 [74.9]	102 [92.8]	224 [82.0]
	No	21 [12.9]	4 [3.6]	25 [9.2]
	Yes	20 [12.2]	4 [3.6]	24 [8.8]
Khat use past 3 months	Never used khat	122 [74.9]	102 [92.7]	224 [82.1]
	No	16 [9.8]	3 [2.7]	19 [6.9]
	Yes	25 [15.3]	5 [4.6]	30 [11.0]
Other drug use past 3 months	No	149 [91.4]	99 [90.0]	248 [90.8]
	Yes	14 [8.6]	11 [10.0]	25 [9.2]
***Disordered Eating behaviour***				
Emotional eating Mean/SD	-	1.9 [0.6]	1.9 [0.6]	1.9 [0.6]
Restrained eating Mean/SD	-	2.0 [0.5]	2.0 [0.6]	2.0 [0.6]
External eating Mean/SD	-	3.0 [0.5]	3.1 [0.5]	3.0 [0.5]

*Used at least one of the following in the past three months: shisha, glue, heroin, cocaine, methamphetamine

STI: Sexually transmitted Infection

WHR [Low risk for cardiovascular complications]: <0.95 or <0.8 for males and females respectively

WHR [High risk for cardiovascular complications]: ≥ 0.95 or ≥ 0.8 for males and females respectively

PEP: Post-exposure prophylaxis

PreP: Pre-exposure prophylaxis

SD: Standard deviation

#### Disordered eating behaviour constructs

Overall, the mean of the three DEB constructs based on all 33 DEBQ items was 1.9 [0.6], 2.0 [0.6] and 3.0 [0.5] for emotional, restrained and external eating respectively (Table 3). The highest mean of emotional eating was recorded among participants who had not used khat in the last 3 months prior to enrolment into the study (2.2 [0.6]). Similarly, the highest mean of restrained and external eating was recorded among participants who had used other drugs (2.3 [0.7]) or marijuana in the past 3 months (3.3 [0.4] (S3 Table).

#### SB latent classes

**Fig 2:**
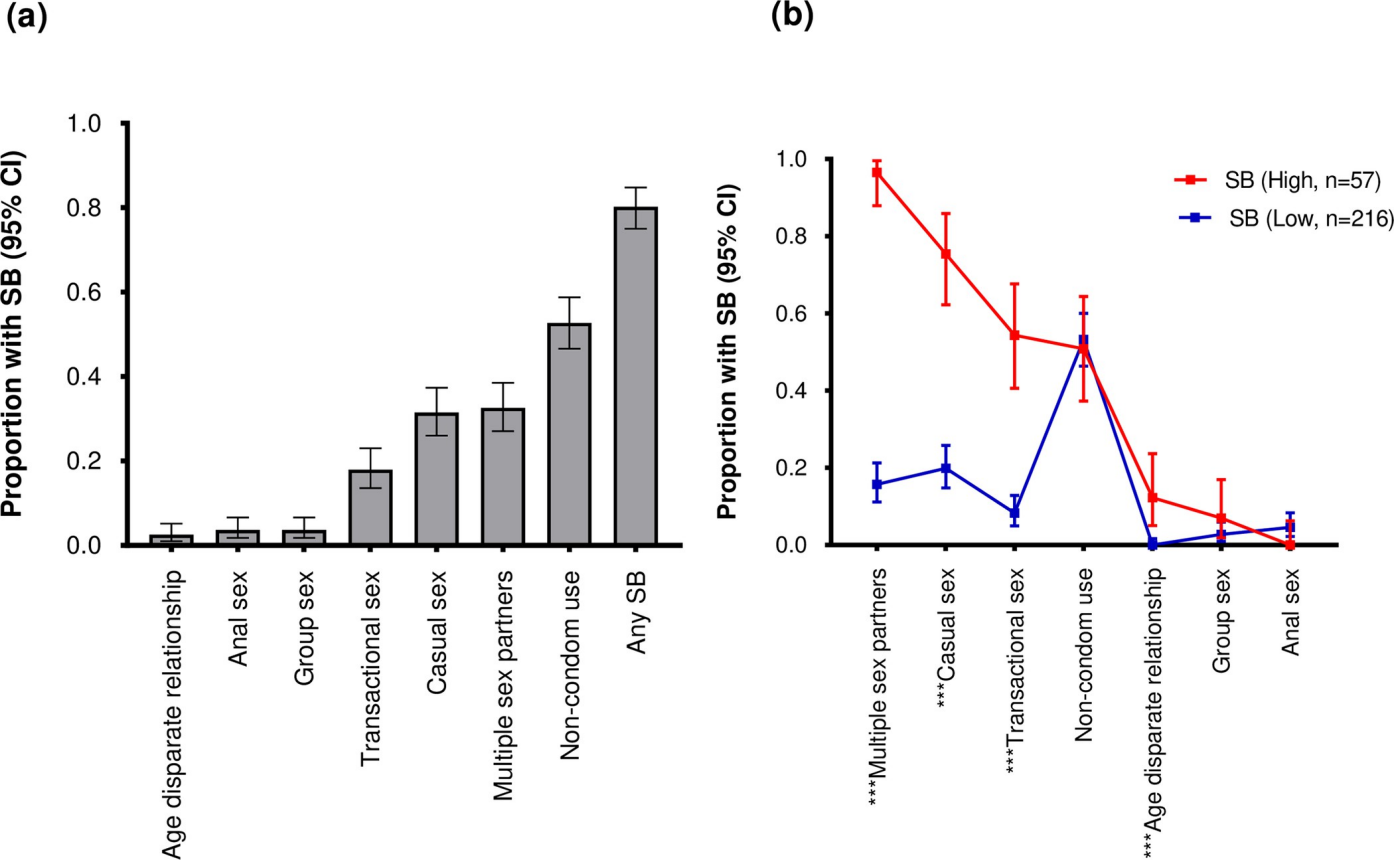
Distribution of participants by SB indicators and SB indicators by SB latent class. (a) Distribution of participants by SB indicators; (b) distribution of SB indicators between participants that were defined to belong to latent high SB class, [SB, Red] with those in latent low SB class, [SB, blue], (Chi-square test p < 0.05 [***], n = 273.

Overall, 219/273 (80.2%) of the participants reported at least one of the seven SB indicators. The most common SB indicator was non-condom use (n = 144 [52.7%]). Based on the LCA, the two-class solution was the best fitting model by AIC and BIC (S4 Table). The two latent classes generated were class one (the low SB class) and class two (the high SB class). Compared to class 2, class 1 comprised the majority of participants (216/273[79.1%] vs 57/273[20.9%]) (Fig 2B). Four out of the seven SB indicators were significantly higher in participants belonging to the latent high SB class compared to those in latent low SB class (Fig 2B). Although group sex was reportedly more in the latent high SB class compared to the latent low SB class, (6.8% vs 2.7%), the difference was not statistically significant (p = 0.130) (Fig 2B).

#### Associations between DEB and SB

Logistic regression was used to explore the associations between DEB represented by the three latent DEB constructs (Emotional eating, restrained eating and external eating) and latent high SB. DEB was not independently associated with latent high SB even after adjusting for socio-demographic, anthropometric and mental health indicators. Specifically, emotional eating (Adjusted odds ratio {AOR [95% confidence interval, CI]: 1.0 [0.9–1.0] p = 0.398), restrained eating (1.0 [0.9–1.1] p = 0.301) and external eating (1.0 [0.8–1.2] p = 0.523) were not independently associated with latent high SB (Table 4). In separate validation analyses, we assessed the association between the three DEB constructs and each of the seven SB indicators included in the LCA. In all the seven models, we did not find an association between DEB and SB except restrained eating which showed an association with anal sex (Adjusted odds ratio {AOR [95% confidence interval {CI}: 1.2 [1.0–1.4]] p = 0.027). However, only 10/273 (3.7% (Fig 2A) of participants had engaged in anal sex hence the suggested association between restrained eating and anal sex needs to be interpreted with caution (S5–S11 Tables).

**Table 4:** Association between disordered eating behaviour and sexual behaviour among of sexually active emerging adults aged 18–24 years attending a tertiary learning institution in Coastal Kenya (N = 273).

Predictor	Category	High SB Class n[%]	Low SB Class n[%]	Crude OR [95% CI]	p-value	Adjusted OR [95% CI]	p-value
Emotional eating	-	23.2 [7.2]	21.1 [7.6]	1.0 [0.9–1.0]	0.065	1.0 [0.9–1.0]	0.398
Restrained eating	-	9.6 [4.0]	9.3 [4.0]	1.0 [0.9–1.0]	0.598	1.0 [0.9–1.1]	0.301
External eating	-	6.8 [1.8]	6.4 [2.0]	1.1 [0.9–1.2]	0.211	1.0 [0.8–1.2]	0.523
Sex	Female	13 [11.8]	97 [88.1]	Ref	Ref	Ref	Ref
	Male	44 [26.9]	119 [73.0]	2.7 [1.4–5.4]	<0.003	0.7 [0.2–2.1]	0.586
Age	18–20	18 [15.9]	95 [84.0]	Ref	Ref	Ref	Ref
	21–24	39 [24.3]	121 [75.6]	1.7 [0.9–3.1]	0.093	1.6 [0.7–3.3]	0.194
Religion	Christian	54 [20.2]	213 [79.7]	Ref	Ref	Ref	Ref
	Muslim	3 [50.0]	3 [50.0]	3.9 [0.7–20.0]	0.098	9.3 [1.21–67.5]	0.027
Perceived chance of getting HIV	Small chance	44 [19.1]	186 [80.8]	Ref	Ref	Ref	Ref
	Great chance	13 [30.2]	30 [69.7]	1.8 [0.8–3.7]	0.104	1.7 [0.7–3.9]	0.192
Ever taken PEP and/or PreP	No	53 [20.1]	210 [79.8]	Ref	Ref	Ref	Ref
	Yes	4 [40.0]	6 [60.0]	2.6 [0.7–9.6]	0.143	1.4 [0.3–6.1]	0.610
Ever gambled	No	20 [15.3]	110 [84.6]	Ref	Ref	Ref	Ref
	Yes	37 [25.8]	106 [74.1]	1.9 [1.0–3.5]	0.035	1.2 [0.5–2.8]	0.610
WHR	Low risk	52 [24.0]	164 [75.9]	Ref	Ref	Ref	Ref
	High risk	5 [8.7]	52 [91.2]	0.3 [0.1–0.7]	0.016	0.2 [0.0–1.0]	0.067
Binge drinking last 3 months	Did not drink last 3 months	21 [14.3]	125 [85.6]	Ref	Ref	Ref	Ref
	No	22 [34.0]	72 [76.6]	1.8 [0.9–3.5]	0.078	1.6 [0.6–3.7]	0.259
	Yes	14 [42.4]	19 [57.5]	4.3 [1.9–10.0]	<0.001	3.3 [1.1–10.3]	0.031
Marijuana use last 3 months	Never used marijuana in life time	33 [16.5]	167 [83.5]	Ref	Ref	Ref	Ref
	No	8 [38.1]	13 [61.9]	3.1 [1.1–8.1]	0.020	1.9 [0.6–6.1]	0.251
	Yes	16 [30.7]	36 [69.2]	2.2 [1.1–4.5]	0.023	0.9 [0.3–2.6]	0.930
Tobacco use last 3 months	Never used tobacco in life time	42 [18.7]	182 [81.2]	Ref	Ref	Ref	Ref
	No	6 [24.0]	19 [76.0]	1.3 [0.5–3.6]	0.529	0.7 [0.2–2.3]	0.594
	Yes	9 [37.5]	15 [62.5]	2.6 [1.0–6.3]	0.036	1.4 [0.4–5.3]	0.536
Chewed khat last 3 months	Never chewed khat in life time	42 [18.7]	182 [81.2]	Ref	Ref	Ref	Ref
	No	5 [26.3]	14 [73.6]	1.5 [0.5–4.5]	0.426	0.8 [0.2–2.8]	0.765
	Yes	10 [33.3]	20 [66.6]	2.1 [0.9–4.9]	0.068	0.6 [0.2–2.0]	0.463
Younger age at sexual debut	No	22 [12.9]	148 [87.0]	Ref	Ref	Ref	Ref
	Yes	35 [33.9]	68 [66.0]	3.4 [1.8–6.3]	<0.001	3.0 [1.4–6.2]	0.003

WHR [High risk for cardiovascular complications]: ≥ 0.95 or ≥ 0.8 for males and females respectively

PEP: Post-exposure prophylaxis

PreP: Pre-exposure prophylaxis

## Discussion

Contrary to our hypothesis, our findings do not suggest an association between any of the three DEB constructs (emotional, restrained and external eating) and SB in this study sample. We had hypothesised that DEB would be associated with SB as reported in previous studies [30, 31, 57, 58]. We propose three explanations for the absence of association. First, most studies reporting associations between DEB and SB were conducted in high income countries [30, 31, 57, 58]. It is possible that the socio-economic differences between high-income countries (where most of the studies reporting an association between DEB and SB were conducted) and low- and middle-income countries (where the current study was conducted) could explain the differences in the findings. Specifically, there is an increased emphasis on thinness in high-income countries. Such emphasis could impact body perception and eventually eating habits leading to DEB [59]. It is not surprising therefore that the mean of DEB in our sample is lower compared to estimates from high income countries. Specifically, the mean of emotional eating in our sample was 1.9 [0.6] compared with 6.4 [2.6] reported amongst 961 college students aged 18–25 years in Florida, USA [60]. One potential explanation for the increased emphasis on thinness in high-income countries is social pressure. Specifically, social pressure regarding thinness and attractiveness, internalization of societal beauty ideals and body dissatisfaction [61] which have been shown to be high in high-income countries [62]. Emerging adults especially young women, are exposed to societal messages about the physical characteristics associated with being beautiful, the importance of being thin, and how being attractive is seen as a characteristic of being successful in life, relationships and career [63]. Because of the discrepancy between the actual body outlook of emerging adults and what they consider to be the ideal body image, this is thought to lead to body image concerns and eventually DEB [64]. Apart from social pressure, another aspect of the sociocultural model explaining the increased emphasis on thinness in high-income countries is the influence of modern media which people have easy access to compared to low- and middle-income countries [59]. Even with increasing urbanization, media access in low- and middle-income countries remains comparatively lower. The disparity in media access, and consequently the influences of media, between the two settings imply a variation in societal preference for thinness and may explain the lack of association between DEB and SB in low- and middle-income countries whereas an association has been found in high-income countries.

Secondly, we used the DEBQ tool to assess DEB. Since the tool has not been validated for use in the local Kenyan context, this may partially explain the lack of association with SB. Although the DEBQ has been found to be valid (spearman correlation coefficient >0.30 and Cronbach’s alpha ≥0.70) among university students in Brazil [65], a middle-income country, it remains unclear if the same may apply in a low- and middle-income African setting like Kenya.

Finally, it is possible that there simply is no association between DEB and SB in this population similar to findings from a study among women in USA that suggests lack of an association between DEB and parity [58].

Our findings imply that in low- and middle-income countries like Kenya, interventions targeted at DEB among emerging adults towards controlling SB are unnecessary since our findings suggest lack of an association between the two. However, our results need to be interpreted with caution given that some studies have reported an association between DEB and SB as well as other risky behaviors including alcohol use [66], tobacco use [67] and illicit drug use. Further, DEB may adversely impact other health indicators including mental health disorders in high-income settings [68], though this has not been extensively studied in low- and middle-income countries and warrants further investigations.

### Study limitations

First, we collected data on binge eating and controlled for it in our analysis. However, we did not collect data on similar eating behaviors like anorexia and bulimia. This may have negatively impacted our ability to disentangle the relationship between DEB and SB which was not existent in our findings. Secondly, we used the DEBQ tool, which has not been validated for the local context, and may have resulted to the lack of an association with SB. Indeed, our study suggests that the DEBQ is valid and reliable for use among Kenyan emerging adults (Cronch bach alpha 0.8770, 0.8002 and 0.7374 for the three latent factors (Emotional, restrained and external eating respectively). Further, while it is true that the DEBQ has not been validated in sSA, specifically Kenya, Brazil, where the tool has been validated [65], is a middle-income country like Kenya [69]. Apart from Brazil, the tool has also been found to be valid among Malaysian university students (Cronch bach alpha 0.8), another middle-income country [70]. Countries classified as middle income share similar demographics especially on economic indicators [69]. This being the case, it is not expected that there would be major differences in performance of the tool between the two settings. Thirdly, the associations between DEB and SB may be mediated by body perception, an important factor not assessed in our study. Fourthly, the use of non-probability sampling and enrolment of participants from one site may limit the generalizability of our findings and the variability of the participants characteristics. However, the homogenous nature of the student population may have reduced the impact of the expected selection bias. Fourthly, we used a sample size of 273 which may be considered small. A sample size of 300 is considered adequate to run an LCA but smaller sample sizes may be used when a few outcome indicators are involved [53, 71]. Given some studies have used more than 20 indicators, the 7 used in this study were considered few hence justifying the sample size of 273. Fifth, the use of a cross sectional design prevents us from establishing causal relationships between DEB and SB, although no association was found. Sixth, the mean WHR obtained by assessor one differed from that of assessor two which may have impacted our analysis. However, none of the three DEB constructs showed an association with latent high SB even after excluding WHR from the multivariable logistic regression model (emotional eating p = 0.353; restrained eating p = 0.394; external eating p = 0.591). Last, our study assessed SBs, including multiple sex partners and qualified them risky. Their consideration as risky is informed by qualifications from previous studies [21, 40–45], and is in no way intended to stigmatise the said SBs.

### Conclusion

In conclusion, there was no significant association between DEB and latent high SB. Our findings suggest that in low- and middle-income countries like Kenya, interventions targeted at DEB among emerging adults towards controlling SB are unnecessary.

## Supporting information

S1 Checklist STROBE statement—checklist of items that should be included in reports of observational studies. (DOCX)

S1 Table Correlation between eating behavior constructs in final model (n = 273). (DOCX)

S2 Table Fit statistics of final DEBQ model (n = 273). (DOCX)

S3 Table Socio-demographic, clinical and psychosocial characteristics of emerging adults aged 18–24 years attending a tertiary learning institution in Coastal Kenya aggregated by eating behaviour constructs (N = 273). (DOCX)

S4 Table Model fit statistics for one class model and two class model in the latent class analysis (n = 273). (DOCX)

S5 Table Associations between disordered eating behaviour and non-condom use among emerging adults aged 18–24 years attending a tertiary institution of learning in Coastal Kenya (n = 273). (DOCX)

S6 Table Associations between disordered eating behaviour and multiple sexual partnerships among emerging adults aged 18–24 years attending a tertiary institution of learning in Coastal Kenya (n = 273). (DOCX)

S7 Table Associations between disordered eating behaviour and casual sex among emerging adults aged 18–24 years attending a tertiary institution of learning in Coastal Kenya (n = 273). (DOCX)

S8 Table Associations between disordered eating behaviour and transactional sex among emerging adults aged 18–24 years attending a tertiary institution of learning in Coastal Kenya (n = 273). (DOCX)

S9 Table Associations between disordered eating behaviour and group sex among emerging adults aged 18–24 years attending a tertiary institution of learning in Coastal Kenya (n = 273). (DOCX)

S10 Table Associations between disordered eating behaviour and anal sex among emerging adults aged 18–24 years attending a tertiary institution of learning in Coastal Kenya (n = 273). (DOCX)

S11 Table Associations between disordered eating behaviour and age-disparate relationships among emerging adults aged 18–24 years attending a tertiary institution of learning in Coastal Kenya (n = 273). (DOCX)
