# Supplementary material for: Associations between disordered eating behaviour and sexual behaviour amongst emerging adults attending a tertiary education institution in Coastal Kenya
Source: PLoS One. 2024 Jun 11;19(6):e0301436. doi: 10.1371/journal.pone.0301436 (PMC11166344; doi:10.1371/journal.pone.0301436)
Supplement: S1 Table — (DOCX) [file pone.0301436.s002.docx]

**S1 Table: Correlation between eating behavior constructs in final model (n=273)**

| **Latent variables** | Emotional eating | Restrained eating | External eating |
| --- | --- | --- | --- |
| Emotional eating | 1.0000 | 0.0533 | 0.1295 |
| Restrained eating | **0.0533** | 1.0000 | -0.0636 |
| External eating | **0.1295** | **-0.0636** | 1.0000 |
