# Supplementary material for: Associations between disordered eating behaviour and sexual behaviour amongst emerging adults attending a tertiary education institution in Coastal Kenya
Source: PLoS One. 2024 Jun 11;19(6):e0301436. doi: 10.1371/journal.pone.0301436 (PMC11166344; doi:10.1371/journal.pone.0301436)
Supplement: S2 Table — (DOCX) [file pone.0301436.s003.docx]

**S2 Table: Fit statistics of final DEBQ model (n=273)**

|  | **(TLI)** | **(CFI)** | **RMSEA** | **RMSR** |
| --- | --- | --- | --- | --- |
| Emotional eating | 0.917 | 0.934 | 0.076 | 0.045 |
| Restrained eating | 0.963 | 0.982 | 0.074 | 0.030 |
| External eating | 1.000 | 1.000 | 0.000 | 0.000 |

TLI- Tucker-Lewis index

CFI- Comparative fit index

RMSEA-Root mean square error of approximation

RMSR- Root mean square residual
