# Supplementary material for: Associations between disordered eating behaviour and sexual behaviour amongst emerging adults attending a tertiary education institution in Coastal Kenya
Source: PLoS One. 2024 Jun 11;19(6):e0301436. doi: 10.1371/journal.pone.0301436 (PMC11166344; doi:10.1371/journal.pone.0301436)
Supplement: S3 Table — (DOCX) [file pone.0301436.s004.docx]

**S3 Table: Socio-demographic, clinical and psychosocial characteristics of emerging adults aged 18-24 years attending a tertiary learning institution in Coastal Kenya aggregated by eating behaviour constructs (N=273)**

| **Characteristics** | **Category** | **Emotional eating*** | **Restrained eating*** | **External eating*** |
| --- | --- | --- | --- | --- |
|  |  | **M [SD]** | **M [SD]** | **M [SD]** |
| ***Socio-demographic and clinical indicators*** |  |  |  |  |
| Age group [Years] | 18 – 20 | 1.9 [0.7] | 2.0 [0.6] | 3.1 [0.6] |
|  | 21 - 24 | 1.9 [0.6] | 2.1 [0.6] | 3.0 [0.5] |
| Year of study | Year 1 & 2 | 1.9 [0.7] | 2.0 [0.6] | 2.9 [0.6] |
|  | Year 3 & 4 | 1.9 [0.6] | 2.1 [0.6] | 3.1 [0.5] |
| Study program was your first choice | No | 2.0 [0.7] | 2.2 [0.7] | 3.0 [0.6] |
|  | Yes | 1.9 [0.6] | 2.0 [0.5] | 3.0 [0.5] |
| Ever been tested for HIV | No | 1.9 [0.7] | 2.0 [0.6] | 3.0 [0.6] |
|  | Yes | 2.0 [0.6] | 2.1 [0.6] | 3.0 [0.5] |
| Have children? | No | 1.9 [0.6] | 2.0 [0.6] | 3.0 [0.5] |
|  | Yes | 2.0 [0.6] | 1.8 [0.3] | 3.2 [0.7] |
| Parents alive? | Both parents alive | 2.0 [0.6] | 2.0 [0.6] | 3.0 [0.5] |
|  | One or both parents dead | 1.8 [0.5] | 2.0 [0.7] | 2.8 [0.5] |
| Living arrangement | In campus | 1.8 [0.7] | 2.0 [0.5] | 2.9 [0.5] |
|  | Outside campus | 1.9 [0.6] | 2.1 [0.6] | 3.0 [0.6] |
| Religion | Catholic | 2.1 [0.6] | 2.0 [0.5] | 3.0 [0.5] |
|  | Protestant or other Christian | 1.9 [0.6] | 2.0 [0.6] | 3.0 [0.5] |
|  | Muslim | 1.8 [0.4] | 2.0 [0.7] | 3.2 [0.4] |
| STI symptoms past 3 months | No | 2.0 [0.6] | 2.0 [0.6] | 3.0 [0.5] |
|  | Yes | 1.9 [0.5] | 2.1 [0.6] | 3.1 [0.5] |
| Perceived chances of getting HIV | Small chance | 1.9 [0.6] | 2.0 [0.6] | 3.0 [0.6] |
|  | Great chance | 2.0 [0.7] | 2.0 [0.5] | 3.1 [0.4] |
| Ever taken PEP and/or PreP | No | 1.9 [0.6] | 2.0 [0.6] | 3.0 [0.5] |
|  | Yes | 2.1 [0.6] | 2.1 [0.4] | 2.8 [0.3] |
| Ever taken part in gambling | No | 1.9 [0.7] | 2.0 [0.6] | 3.1 [0.5] |
|  | Yes | 1.9 [0.6] | 2.0 [0.5] | 3.0 [0.5] |
| Seriously injured past 3 months | No | 1.9 [0.6] | 2.0 [0.6] | 3.1 [0.6] |
|  | Yes | 2.1 [0.5] | 2.1 [0.6] | 3.0 [0.4] |
| Binge eating ever | No | 1.9 [0.5] | 2.0 [0.5] | 2.9 [0.5] |
|  | Yes | 2.0 [0.7] | 2.0 [0.6] | 3.1 [0.5] |
| ***Anthropometric indicators*** |  |  |  |  |
| Body Mass Index categories | Low [<18.5] | 1.9 [0.5] | 2.0 [0.4] | 3.2 [0.4] |
|  | Normal [18.5 – 25] | 1.9 [0.6] | 2.0 [0.5] | 3.0 [0.5] |
|  | High [ >25] | 1.9 [0.5] | 2.5 [0.7] | 2.9 [0.6] |
| Waist-Hip Ratio categories | Low risk | 1.9 [0.7] | 2.0 [0.6] | 3.0 [0.5] |
|  | High risk | 1.9 [0.6] | 2.1 [0.6] | 3.1 [0.6] |
| ***Mental health indicators*** |  |  |  |  |
| Binge drinking past 3 months | No alcohol last 3 months | 1.8 [0.6] | 2.0 [0.6] | 2.9 [0.5] |
|  | No | 2.0 [0.7] | 2.0 [0.6] | 3.1 [0.5] |
|  | Yes | 2.1 [0.6] | 2.1 [0.5] | 3.0 [0.7] |
| Marijuana use past 3 months | Never used marijuana | 1.9 [0.7] | 2.0 [0.6] | 2.9 [0.5] |
|  | No | 1.8 [0.6] | 1.9 [0.5] | 3.0 [0.7] |
|  | Yes | 2.1 [0.5] | 2.0 [0.7] | 3.3 [0.4] |
| Tobacco use past 3 months | Never used tobacco | 1.9 [0.6] | 2.0 [0.6] | 3.0 [0.5] |
|  | No | 1.9 [0.5] | 2.0 [0.7] | 3.1 [0.4] |
|  | Yes | 2.1 [0.6] | 1.9 [0.5] | 3.2 [0.6] |
| Khat use past 3 months | Never used khat | 1.9 [0.6] | 2.0 [0.6] | 3.0 [0.5] |
|  | No | 2.2 [0.6] | 1.8 [0.4] | 3.0 [0.4] |
|  | Yes | 2.1 [0.5] | 2.1 [0.7] | 3.2 [0.5] |
| Other drug use past 3 months** | No | 1.9 [0.6] | 2.0 [0.6] | 3.0 [0.5] |
|  | Yes | 2.0 [0.5] | 2.3 [0.7] | 3.0 [0.6] |

STI: Sexually transmitted Infection

WHR [Low risk for cardiovascular complications]: <0.95 or <0.8 for males and females respectively

WHR [High risk for cardiovascular complications]: ≥ 0.95 or ≥ 0.8 for males and females respectively

PEP: Post-exposure prophylaxis

PreP: Pre-exposure prophylaxis

*Eating behaviour constructs are derived from the original 33 DEBQ items

**Used at least one of the following in the past three months: shisha, glue, heroin, cocaine, methamphetamine
