# Supplementary material for: Associations between disordered eating behaviour and sexual behaviour amongst emerging adults attending a tertiary education institution in Coastal Kenya
Source: PLoS One. 2024 Jun 11;19(6):e0301436. doi: 10.1371/journal.pone.0301436 (PMC11166344; doi:10.1371/journal.pone.0301436)
Supplement: S4 Table — (DOCX) [file pone.0301436.s005.docx]

**S4 Table: Model fit statistics for one class model and two class model in the latent class analysis (n=273)**

| **Number of classes** | **AIC** | **BIC** |
| --- | --- | --- |
| 1 | 1570.119 | 1595.386 |
| 2 | 1536.481 | 1590.623 |

AIC: Akaike’s Information Criterion

BIC: Bayesian Information Criterion
