# Supplementary material for: Associations between disordered eating behaviour and sexual behaviour amongst emerging adults attending a tertiary education institution in Coastal Kenya
Source: PLoS One. 2024 Jun 11;19(6):e0301436. doi: 10.1371/journal.pone.0301436 (PMC11166344; doi:10.1371/journal.pone.0301436)
Supplement: S5 Table — (DOCX) [file pone.0301436.s006.docx]

**S5 Table: Associations between disordered eating behaviour and non-condom use among emerging adults aged 18 – 24 years attending a tertiary institution of learning in Coastal Kenya (n = 273)**

| **Particulars** | **Category** | **Didn’t use condom n [%]** | **Used condom n [%]** | **Crude OR [95% CI]** | **p-value** | **Adjusted OR [95% CI]** | **p-value** |
| --- | --- | --- | --- | --- | --- | --- | --- |
| Emotional eating [M/SD] | - | 21.8 [7.1] | 21.3 [8.1] | 1.0 [0.9 – 1.0] | 0.546 | 1.0 [0.9 – 1.0] | 0.437 |
| Restrained eating [M/SD] | - | 9.3 [4.0] | 9.5 [3.9] | 0.9 [0.9 – 1.0] | 0.684 | 0.9 [0.9 – 1.0] | 0.990 |
| External eating [M/SD] | - | 6.6 [2.0] | 6.4 [1.9] | 1.0 [0.9 – 1.2] | 0.476 | 0.9 [0.8 – 1.1] | 0.921 |
| Sex | Female | 69 [62.7] | 41 [37.2] | Ref | Ref | Ref | Ref |
|  | Male | 75 [46.0] | 88 [53.9] | 0.5 [0.3 – 0.8] | 0.007 | 0.3 [0.1 – 0.8] | 0.020 |
| Year of study | Year 1 and 2 | 75 [48.4] | 80 [51.6] | Ref | Ref | Ref | Ref |
|  | Year 3 and 4 | 69 [58.5] | 49 [41.5] | 1.5 [0.9 – 2.4] | 0.099 | 1.4 [0.8 – 2.5] | 0.211 |
| Having a child | No | 128 [50.2] | 127 [49.8] | Ref | Ref | Ref | Ref |
|  | Yes | 16 [88.9] | 2 [11.1] | 7.9 [1.8 – 35.2] | 0.006 | 8.7 [1.7 – 42.9] | 0.007 |
| Syndromic STI* last 3 months | No | 84 [48.8] | 88 [51.1] | Ref | Ref | Ref | Ref |
|  | Yes | 60 [59.4] | 41 [40.5] | 1.5 [0.9 – 2.5] | 0.092 | 0.8 [0.4 – 1.6] | 0.626 |
| Perceived chance of contracting HIV | Small chance | 113 [49.1] | 117 [50.8] | Ref | Ref | Ref | Ref |
|  | Great chance | 31 [72.1] | 12 [27.9] | 2.7 [1.3 – 5.5] | 0.007 | 2.8 [1.2 – 6.4] | 0.010 |
| Waist for Hip Ratio (WHR) | Low risk | 109 [50.5] | 107 [49.5] | Ref | Ref | Ref | Ref |
|  | High risk | 35 [61.4] | 22 [38.6] | 1.6 [0.9 – 2.8] | 0.143 | 0.7 [0.3 – 1.8] | 0.583 |
| Binge drinking last 3 months | Did not drink last 3 months | 79 [54.1] | 67 [45.8] | Ref | Ref | Ref | Ref |
|  | No | 43 [45.7] | 51 [54.2] | 0.7 [0.4 – 1.2] | 0.206 | 0.4 [0.2 – 0.9] | 0.042 |
|  | Yes | 22 [66.7] | 11 [33.3] | 1.7 [0.8 – 3.8] | 0.192 | 0.9 [0.3 – 2.5] | 0.875 |
| Marijuana use last 3 months | Never used marijuana in life time | 99 [49.5] | 101 [50.5] | Ref | Ref | Ref | Ref |
|  | No | 14 [66.7] | 7 [33.3] | 2.0 [0.8 – 5.3] | 0.141 | 3.4 [1.1 – 10.2] | 0.026 |
|  | Yes | 31 [59.6] | 21 [40.3] | 1.5 [0.8 – 2.7] | 0.195 | 1.3 [0.5 – 3.2] | 0.551 |
| Tobacco use last 3 months | Never used tobacco in life time | 111 [49.6] | 113 [50.4] | Ref | Ref | Ref | Ref |
|  | No | 13 [52.0] | 12 [48.0] | 1.1 [0.5 – 2.5] | 0.817 | 1.1 [0.4 – 3.1] | 0.784 |
|  | Yes | 20 [83.3] | 4 [16.6] | 5.1 [1.7 – 15.4] | 0.004 | 4.8 [1.2 – 18.7] | 0.021 |
| Chewed khat last 3 months | Never chewed khat in life time | 111 [49.6] | 113 [50.4] | Ref | Ref | Ref | Ref |
|  | No | 12 [63.2] | 7 [36.8] | 1.7 [0.7 – 4.6] | 0.260 | 1.8 [0.5 – 6.0] | 0.279 |
|  | Yes | 21 [70.0] | 9 [30.0] | 2.4 [1.0 – 5.4] | 0.040 | 2.9 [1.0 – 8.5] | 0.042 |
| Younger age at sexual debut | No | 95 [55.9] | 75 [44.1] | Ref | Ref | Ref | Ref |
|  | Yes | 49 [47.6] | 54 [52.4] | 0.7 [0.4 – 1.2] | 0.183 | 0.5 [0.3 – 1.1] | 0.098 |

*STI – Sexually transmitted infection
