# Supplementary material for: Associations between disordered eating behaviour and sexual behaviour amongst emerging adults attending a tertiary education institution in Coastal Kenya
Source: PLoS One. 2024 Jun 11;19(6):e0301436. doi: 10.1371/journal.pone.0301436 (PMC11166344; doi:10.1371/journal.pone.0301436)
Supplement: S6 Table — (DOCX) [file pone.0301436.s007.docx]

**S6 Table: Associations between disordered eating behaviour and multiple sexual partnerships among emerging adults aged 18 – 24 years attending a tertiary institution of learning in Coastal Kenya (n = 273)**

| **Particulars** | **Category** | **MSP n [%]** | **No MSP n [%]** | **Crude OR [95% CI]** | **p-value** | **Adjusted OR [95% CI]** | **p-value** |
| --- | --- | --- | --- | --- | --- | --- | --- |
| Emotional eating [M/SD] | - | 23.1 [7.7] | 20.8 [7.4] | 1.0 [1.0 – 1.1] | 0.024 | 1.0 [0.9 – 1.0] | 0.136 |
| Restrained eating [M/SD] | - | 9.4 [4.1] | 9.3 [3.9] | 1.0 [0.9 – 1.0] | 0.911 | 1.0 [0.9 – 1.1] | 0.546 |
| External eating [M/SD] | - | 6.8 [1.9] | 6.4 [2.0] | 1.0 [0.9 – 1.2] | 0.161 | 1.0 [0.8 – 1.1] | 0.845 |
| Sex | Female | 19 [17.2] | 91 [82.7] | Ref | Ref | Ref | Ref |
|  | Male | 70 [42.9] | 93 [57.0] | 3.6 [2.0 – 6.4] | <0.001 | 1.0 [0.3 – 3.0] | 0.902 |
| Age [Years] | 18 - 20 | 31 [27.4] | 82 [72.5] | Ref | Ref | Ref | Ref |
|  | 21 - 24 | 58 [36.2] | 102 [63.7] | 1.5 [0.8 – 2.5] | 0.127 | 1.2 [0.6 – 2.2] | 0.558 |
| Tested for HIV ever | No | 63 [29.7] | 149 [70.2] | Ref | Ref | Ref | Ref |
|  | Yes | 26 [42.6] | 35 [57.3] | 1.7 [0.9 – 3.1] | 0.060 | 1.8 [0.8 – 3.7] | 0.103 |
| Religion | Christian | 85 [31.8] | 182 [68.1] | Ref | Ref | Ref | Ref |
|  | Muslim | 4 [66.6] | 2 [33.3] | 4.2 [0.7 – 23.8] | 0.097 | 11.1 [1.3 – 92.3] | 0.025 |
| Syndromic STI* last 3 months | No | 62 [36.0] | 110 [63.9] | Ref | Ref | Ref | Ref |
|  | Yes | 27 [26.7] | 74 [73.2] | 0.6 [0.3 – 1.1] | 0.114 | 1.3 [0.6 – 2.8] | 0.487 |
| Perceived chance of contracting HIV | Small chance | 69 [30.0] | 161 [70.0] | Ref | Ref | Ref | Ref |
|  | Great chance | 20 [46.5] | 23 [53.4] | 2.0 [1.0 – 3.9] | 0.036 | 1.9 [0.9 – 4.3] | 0.088 |
| Gambling ever | No | 28 [21.5] | 102 [78.4] | Ref | Ref | Ref | ref |
|  | Yes | 61 [42.6] | 82 [57.3] | 2.7 [1.5 – 4.6] | <0.001 | 1.9 [0.9 – 3.9] | 0.085 |
| Waist for Hip Ratio (WHR) | Low risk | 82 [37.9] | 134 [62.0] | Ref | Ref | Ref | Ref |
|  | High risk | 7 [12.2] | 50 [87.7] | 0.2 [0.1 – 0.5] | 0.001 | 0.2 [0.1 – 0.8] | 0.027 |
| Binge drinking last 3 months | Did not drink last 3 months | 32 [21.9] | 114 [78.0] | Ref | Ref | Ref | Ref |
|  | No | 39 [41.4] | 55 [58.5] | 2.5 [1.4 – 4.4] | 0.001 | 1.3 [0.6 – 2.8] | 0.399 |
|  | Yes | 18 [54.5] | 15 [45.4] | 4.2 [1.9 – 9.4] | <0.001 | 2.2 [0.7 – 6.4] | 0.145 |
| Marijuana use last 3 months | Never used marijuana in life time | 49 [24.5] | 151 [75.5] | Ref | Ref | Ref | Ref |
|  | No | 11 [52.3] | 10 [47.6] | 3.3 [1.3 – 8.4] | 0.009 | 2.1 [0.7 – 6.2] | 0.180 |
|  | Yes | 29 [55.7] | 23 [44.2] | 3.8 [2.0 – 7.3] | <0.001 | 2.6 [1.0 – 6.6] | 0.042 |
| Tobacco use last 3 months | Never used tobacco in life time | 63 [28.1] | 161 [71.8] | Ref | Ref | Ref | Ref |
|  | No | 14 [56.0] | 11 [44.0] | 3.2 [1.4 – 7.5] | 0.006 | 1.6 [0.5 – 4.8] | 0.327 |
|  | Yes | 12 [50.0] | 12 [50.0] | 2.5 [1.0 – 5.9] | 0.031 | 0.7 [0.2 – 2.3] | 0.566 |
| Chewed khat last 3 months | Never chewed khat in life time | 64 [28.5] | 160 [71.4] | Ref | Ref | Ref | Ref |
|  | No | 8 [42.1] | 11 [57.8] | 1.8 [0.6 – 4.7] | 0.220 | 0.6 [0.2 – 2.0] | 0.456 |
|  | Yes | 17 [56.6] | 13 [43.3] | 3.2 [1.5 – 7.1] | 0.003 | 0.8 [0.3 – 2.5] | 0.832 |
| Younger age at sexual debut | No | 41 [24.1] | 129 [75.8] | Ref | Ref | Ref | Ref |
|  | Yes | 48 [46.6] | 55 [53.4] | 2.7 [1.6 – 4.6] | <0.001 | 1.7 [0.9 – 3.2] | 0.098 |

*STI – Sexually transmitted infection
