# Supplementary material for: Associations between disordered eating behaviour and sexual behaviour amongst emerging adults attending a tertiary education institution in Coastal Kenya
Source: PLoS One. 2024 Jun 11;19(6):e0301436. doi: 10.1371/journal.pone.0301436 (PMC11166344; doi:10.1371/journal.pone.0301436)
Supplement: S7 Table — (DOCX) [file pone.0301436.s008.docx]

**S7 Table: Associations between disordered eating behaviour and casual sex among emerging adults aged 18 – 24 years attending a tertiary institution of learning in Coastal Kenya (n = 273)**

| **Particulars** | **Category** | **Casual sex n [%]** | **No casual sex n [%]** | **Crude OR [95% CI]** | **p-value** | **Adjusted OR [95% CI]** | **p-value** |
| --- | --- | --- | --- | --- | --- | --- | --- |
| Emotional eating [M/SD] | - | 21.8 [7.3] | 21.5 [7.7] | 1.0 [0.9 – 1.0] | 0.779 | 0.9 [0.9 – 1.0] | 0.455 |
| Restrained eating [M/SD] | - | 8.8 [3.7] | 9.6 [4.1] | 0.9 [0.8 – 1.0] | 0.143 | 0.9 [0.8 – 1.0] | 0.315 |
| External eating [M/SD] | - | 6.8 [1.7] | 6.4 [2.0] | 1.1 [0.9 – 1.2] | 0.145 | 1.1 [0.9 – 1.2] | 0.164 |
| Sex | Female | 25 [22.7] | 85 [77.2] | Ref | Ref | Ref | Ref |
|  | Male | 61 [37.4] | 102 [62.5] | 2.0 [1.1 – 3.5] | 0.011 | 0.8 [0.3 – 1.8] | 0.650 |
| Having a child | No | 83 [32.5] | 172 [67.4] | Ref | Ref | Ref | Ref |
|  | Yes | 3 [16.6] | 15 [83.3] | 0.4 [0.1 – 1.4] | 0.173 | 0.3 [0.1 – 1.4] | 0.150 |
| Parents dead | Both parents alive | 73 [33.3] | 146 [66.6] | Ref | Ref | Ref | Ref |
|  | One or both parents dead | 13 [24.0] | 41 [75.9] | 0.6 [0.3 – 1.2] | 0.192 | 0.5 [0.2 – 1.0] | 0.082 |
| Waist for Hip Ratio (WHR) | Low risk | 77 [35.6] | 139 [64.3] | Ref | Ref | Ref | Ref |
|  | High risk | 9 [15.7] | 48 [84.2] | 0.3 [0.1 – 0.7] | 0.005 | 0.3 [0.1 – 0.9] | 0.047 |
| Binge drinking last 3 months | Did not drink last 3 months | 39 [26.7] | 107 [73.2] | Ref | Ref | Ref | Ref |
|  | No | 34 [36.1] | 60 [63.8] | 1.5 [0.8 – 2.7] | 0.121 | 1.2 [0.6 – 2.4] | 0.558 |
|  | Yes | 13 [39.3] | 20 [60.6] | 1.7 [0.8 – 3.9] | 0.151 | 1.2 [0.4 – 3.3] | 0.687 |
| Marijuana use last 3 months | Never used marijuana in life time | 56 [28.0] | 144 [72.0] | Ref | Ref | Ref | Ref |
|  | No | 10 [47.6] | 11 [52.3] | 2.3 [0.9 – 5.8] | 0.068 | 1.7 [0.6 – 4.8] | 0.270 |
|  | Yes | 20 [38.4] | 32 [61.5] | 1.6 [0.8 – 3.0] | 0.145 | 0.8 [0.3 – 1.9] | 0.683 |
| Tobacco use last 3 months | Never used tobacco in life time | 66 [29.4] | 158 [70.5] | Ref | Ref | Ref | Ref |
|  | No | 9 [36.0] | 16 [64.0] | 1.3 [0.5 – 3.2] | 0.500 | 0.7 [0.2 – 2.0] | 0.602 |
|  | Yes | 11 [45.8] | 13 [54.1] | 2.0 [0.8 – 4.7] | 0.105 | 1.5 [0.5 – 4.5] | 0.438 |
| Younger age at sexual debut | No | 41 [24.1] | 129 [75.8] | Ref | Ref | Ref | Ref |
|  | Yes | 45 [43.6] | 58 [56.3] | 2.4 [1.4 – 4.1] | 0.001 | 2.4 [1.3 – 4.5] | 0.004 |
