# Supplementary material for: Associations between disordered eating behaviour and sexual behaviour amongst emerging adults attending a tertiary education institution in Coastal Kenya
Source: PLoS One. 2024 Jun 11;19(6):e0301436. doi: 10.1371/journal.pone.0301436 (PMC11166344; doi:10.1371/journal.pone.0301436)
Supplement: S8 Table — (DOCX) [file pone.0301436.s009.docx]

**S8 Table: Associations between disordered eating behaviour and transactional sex among emerging adults aged 18 – 24 years attending a tertiary institution of learning in Coastal Kenya (n = 273)**

| **Particulars** | **Category** | **Transactional sex n [%]** | **No transactional sex n [%]** | **Crude OR [95% CI]** | **p-value** | **Adjusted OR [95% CI]** | **p-value** |
| --- | --- | --- | --- | --- | --- | --- | --- |
| Emotional eating [M/SD] | - | 22.5 [7.0] | 21.3 [7.7] | 1.0 [0.9 – 1.0] | 0.321 | 1.0 [0.9 – 1.0] | 0.726 |
| Restrained eating [M/SD] | - | 9.2 [3.5] | 9.4 [4.1] | 0.9 [0.9 – 1.0] | 0.737 | 0.9 [0.9 – 1.0] | 0.957 |
| External eating [M/SD] | - | 6.6 [1.9] | 6.5 [2.0] | 1.0 [0.8 – 1.1] | 0.791 | 0.9 [0.8 – 1.1] | 0.972 |
| Sex | Female | 12 [10.9] | 98 [89.0] | Ref | Ref | Ref | Ref |
|  | Male | 37 [22.7] | 126 [77.3] | 2.3 [1.1 – 4.8] | 0.015 | 0.6 [0.1 – 2.3] | 0.526 |
| Study program first choice | No | 19 [22.6] | 65 [77.3] | Ref | Ref | Ref | Ref |
|  | Yes | 30 [15.8] | 159 [84.1] | 0.6 [0.3 – 1.2] | 0.182 | 0.6 [0.3 – 1.4] | 0.324 |
| Syndromic STI* last 3 months | No | 38 [22.0] | 134 [77.9] | Ref | Ref | Ref | Ref |
|  | Yes | 11 [10.8] | 90 [89.1] | 0.4 [0.2 – 0.8] | 0.022 | 0.5 [0.2 – 1.4] | 0.260 |
| Gambling ever | No | 18 [13.8] | 112 [86.1] | Ref | Ref | Ref | Ref |
|  | Yes | 31 [21.6] | 112 [78.3] | 1.7 [0.9 – 3.2] | 0.094 | 1.1 [0.4 – 2.6] | 0.789 |
| Waist for Hip Ratio (WHR) | Low risk | 44 [20.3] | 172 [79.6] | Ref | Ref | Ref | ref |
|  | High risk | 5 [8.7] | 52 [91.2] | 0.3 [0.1 – 0.9] | 0.049 | 0.5 [0.1 – 1.8] | 0.322 |
| Binge drinking last 3 months | Did not drink last 3 months | 22 [15.0] | 124 [84.9] | Ref | Ref | Ref | Ref |
|  | No | 13 [13.8] | 81 [86.1] | 0.9 [0.4 – 1.8] | 0.791 | 0.3 [0.1 – 1.0] | 0.061 |
|  | Yes | 14 [42.4] | 19 [57.5] | 4.1 [1.8 – 9.4] | 0.001 | 1.2 [0.3 – 3.9] | 0.724 |
| Marijuana use last 3 months | Never used marijuana in life time | 26 [13.0] | 174 [87.0] | Ref | Ref | Ref | Ref |
|  | No | 8 [38.1] | 13 [61.9] | 4.1 [1.5 – 10.8] | 0.004 | 3.4 [1.0 – 11.4] | 0.041 |
|  | Yes | 15 [28.8] | 37 [71.1] | 2.7 [1.3 – 5.6] | 0.007 | 1.7 [0.5 – 5.2] | 0.334 |
| Tobacco use last 3 months | Never used tobacco in life time | 33 [14.7] | 191 [85.2] | Ref | Ref | Ref | Ref |
|  | No | 6 [24.0] | 19 [76.0] | 1.8 [0.6 – 4.9] | 0.232 | 1.2 [0.3 – 4.3] | 0.733 |
|  | Yes | 10 [41.6] | 14 [58.3] | 4.1 [1.6 – 10.0] | 0.002 | 2.1 [0.5 – 7.8] | 0.251 |
| Chewed khat last 3 months | Never chewed khat in life time | 32 [14.2] | 192 [85.7] | Ref | Ref | Ref | Ref |
|  | No | 6 [31.5] | 13 [68.4] | 2.7 [0.9 – 7.8] | 0.054 | 1.6 [0.4 – 5.5] | 0.409 |
|  | Yes | 11 [36.6] | 19 [63.3] | 3.4 [1.5 – 7.9] | 0.003 | 1.6 [0.5 – 5.1] | 0.383 |
| Younger age at sexual debut | No | 20 [11.7] | 150 [88.2] | Ref | Ref | Ref | Ref |
|  | Yes | 29 [28.1] | 74 [71.8] | 2.9 [1.5 – 5.5] | 0.001 | 2.3 [1.0 – 4.9] | 0.029 |

*STI – Sexually transmitted infection
