# Supplementary material for: Associations between disordered eating behaviour and sexual behaviour amongst emerging adults attending a tertiary education institution in Coastal Kenya
Source: PLoS One. 2024 Jun 11;19(6):e0301436. doi: 10.1371/journal.pone.0301436 (PMC11166344; doi:10.1371/journal.pone.0301436)
Supplement: S9 Table — (DOCX) [file pone.0301436.s010.docx]

**S9 Table: Associations between disordered eating behaviour and group sex among emerging adults aged 18 – 24 years attending a tertiary institution of learning in Coastal Kenya (n = 273)**

| **Particulars** | **Category** | **Group sex n [%]** | **No group sex n [%]** | **Crude OR [95% CI]** | **p-value** | **Adjusted OR [95% CI]** | **p-value** |
| --- | --- | --- | --- | --- | --- | --- | --- |
| Emotional eating [M/SD] | - | 24.6 [5.3] | 21.4 [7.6] | 1.0 [0.9 – 1.1] | 0.209 | 1.0 [0.9 – 1.1] | 0.288 |
| Restrained eating [M/SD] | - | 8.5 [3.3] | 9.4 [4.0] | 0.9 [0.7 – 1.1] | 0.473 | 0.8 [0.7 – 1.1] | 0.332 |
| External eating [M/SD] | - | 6.7 [2.2] | 6.5 [2.0] | 1.0 [0.7 – 1.4] | 0.827 | 1.0 [0.7 – 1.5] | 0.794 |
| Has a child | No | 8 [3.1] | 247 [96.8] | Ref | Ref | Ref | Ref |
|  | Yes | 2 [11.1] | 16 [88.8] | 3.8 [0.7 – 19.6] | 0.104 | 5.5 [0.8 – 35.8] | 0.070 |
| Ever taken PEP* or PreP** | No | 8 [3.0] | 255 [96.9] | Ref | Ref | Ref | Ref |
|  | Yes | 2 [20.0] | 8 [80.0] | 7.9 [1.4 – 43.6] | 0.017 | 10.4 [1.3 – 83.3] | 0.026 |
| Gambling ever | No | 7 [5.3] | 123 [94.6] | Ref | Ref | Ref | Ref |
|  | Yes | 3 [2.1] | 140 [97.9] | 0.3 [0.1 – 1.4] | 0.164 | 0.1 [0.0 – 0.9] | 0.048 |
| Binge drinking last 3 months | Did not drink last 3 months | 4 [2.7] | 142 [97.2] | Ref | Ref | Ref | Ref |
|  | No | 3 [3.1] | 91 [96.8] | 1.1 [0.2 – 5.3] | 0.839 | 1.9 [0.3 – 11.7] | 0.482 |
|  | Yes | 3 [9.0] | 30 [90.9] | 3.5 [0.7 – 16.6] | 0.109 | 6.7 [0.8 – 54.3] | 0.072 |
| Marijuana use last 3 months | Never used marijuana in life time | 6 [3.0] | 194 [97.0] | Ref | Ref | Ref | Ref |
|  | No | 2 [9.5] | 19 [90.4] | 3.4 [0.6 – 18.0] | 0.150 | 3.6 [0.5 – 25.6] | 0.194 |
|  | Yes | 2 [3.8] | 50 [96.1] | 1.2 [0.2 – 6.6] | 0.757 | 0.5 [0.1 – 4.6] | 0.580 |

*PEP – Post exposure prophylaxis

**PreP – Pre-exposure prophylaxis
