# Supplementary material for: Associations between disordered eating behaviour and sexual behaviour amongst emerging adults attending a tertiary education institution in Coastal Kenya
Source: PLoS One. 2024 Jun 11;19(6):e0301436. doi: 10.1371/journal.pone.0301436 (PMC11166344; doi:10.1371/journal.pone.0301436)
Supplement: S10 Table — (DOCX) [file pone.0301436.s011.docx]

**S10 Table: Associations between disordered eating behaviour and anal sex among emerging adults aged 18 – 24 years attending a tertiary institution of learning in Coastal Kenya (n = 273)**

| **Particulars** | **Category** | **Anal sex n [%]** | **No anal sex n [%]** | **Crude OR [95% CI]** | **p-value** | **Adjusted OR [95% CI]** | **p-value** |
| --- | --- | --- | --- | --- | --- | --- | --- |
| Emotional eating [M/SD] | - | 25.9 [5.6] | 21.4 [7.6] | 1.0 [0.9 – 1.1] | 0.075 | 1.0 [0.9 – 1.1] | 0.222 |
| Restrained eating [M/SD] | - | 11.7 [4.3] | 9.3 [3.9] | 1.1 [0.9 – 1.2] | 0.073 | 1.2 [1.0 – 1.4] | 0.027 |
| External eating [M/SD] | - | 7.6 [1.5] | 6.5 [2.0] | 1.3 [0.9 – 1.9] | 0.099 | 1.3 [0.8 – 2.1] | 0.188 |
| Sex | Female | 7 [6.3] | 103 [93.6] | Ref | Ref | Ref | Ref |
|  | Male | 3 [1.8] | 160 [98.1] | 0.2 [0.1 – 1.0] | 0.066 | 0.1 [0.0 – 1.4] | 0.096 |
| Year of study | Year 1 and 2 | 8 [5.1] | 147 [94.8] | Ref | Ref | Ref | Ref |
|  | Year 3 and 4 | 2 [1.6] | 116 [98.3] | 0.3 [0.1 – 1.5] | 0.151 | 0.1 [0.0 – 1.1] | 0.070 |
| Gambling ever | No | 8 [6.1] | 122 [93.8] | Ref | Ref | Ref | Ref |
|  | Yes | 2 [1.4] | 141 [98.6] | 0.2 [0.0 – 1.0] | 0.056 | 0.2 [0.0 – 3.0] | 0.259 |
| Serious injury | No | 6 [2.5] | 232 [97.4] | Ref | Ref | Ref | Ref |
|  | Yes | 4 [11.4] | 31 [88.5] | 4.9 [1.3 – 18.6] | 0.017 | 14.5 [2.3 – 90.8] | 0.004 |
| Chewed khat last 3 months | Never chewed khat in life time | 6 [2.6] | 218 [97.3] | Ref | Ref | Ref | Ref |
|  | No | 1 [5.2] | 18 [94.7] | 2.0 [0.2 – 17.6] | 0.526 | 6.5 [0.4 – 92.4] | 0.166 |
|  | Yes | 3 [10.0] | 27 [90.0] | 4.0 [0.9 – 17.0] | 0.058 | 18.3 [1.8 – 181.2] | 0.013 |
| Other drug use last 3 months | No | 7 [2.8] | 241 [97.1] | Ref | Ref | Ref | Ref |
|  | Yes | 3 [12.0] | 22 [88.0] | 4.6 [1.1 – 19.4] | 0.033 | 1.5 [0.2 – 8.7] | 0.593 |
