# Supplementary material for: Associations between disordered eating behaviour and sexual behaviour amongst emerging adults attending a tertiary education institution in Coastal Kenya
Source: PLoS One. 2024 Jun 11;19(6):e0301436. doi: 10.1371/journal.pone.0301436 (PMC11166344; doi:10.1371/journal.pone.0301436)
Supplement: S11 Table — (DOCX) [file pone.0301436.s012.docx]

**S11 Table: Associations between disordered eating behaviour and age-disparate relationships among emerging adults aged 18 – 24 years attending a tertiary institution of learning in Coastal Kenya (n = 273)**

| **Particulars** | **Category** | **Age-disparate n [%]** | **No age-disparate n [%]** | **Crude OR [95% CI]** | **p-value** | **Adjusted OR [95% CI]** | **p-value** |
| --- | --- | --- | --- | --- | --- | --- | --- |
| Emotional eating [M/SD] | - | 20.7 [6.1] | 21.6 [7.6] | 0.9 [0.8 – 1.0] | 0.752 | 0.9 [0.8 – 1.1] | 0.755 |
| Restrained eating [M/SD] | - | 8.2 [2.2] | 9.4 [4.0] | 0.9 [0.7 – 1.1] | 0.461 | 0.9 [0.6 – 1.2] | 0.577 |
| External eating [M/SD] | - | 6.0 [2.5] | 6.5 [1.9] | 0.8 [0.6 – 1.2] | 0.452 | 0.8 [0.5 – 1.3] | 0.627 |
| Perceived chance of contracting HIV | Small chance | 4 [1.7] | 226 [98.2] | Ref | Ref | Ref | Ref |
|  | Great chance | 3 [6.9] | 40 [93.0] | 4.2 [0.9 – 19.6] | 0.065 | 5.2 [0.8 – 34.0] | 0.080 |
| Ever taken PEP* or PreP** | No | 6 [2.2] | 257 [97.7] | Ref | Ref | Ref | Ref |
|  | Yes | 1 [10.0] | 9 [90.0] | 4.7 [0.5 – 43.7] | 0.168 | 6.1 [0.5 – 75.7] | 0.155 |
| Binge drinking last 3 months | Did not drink last 3 months | 1 [0.6] | 145 [99.3] | Ref | Ref | Ref | Ref |
|  | No | 3 [3.1] | 91 [96.8] | 4.7 [0.4 – 46.6] | 0.178 | 8.2 [0.5 – 115.7] | 0.119 |
|  | Yes | 3 [9.0] | 30 [90.9] | 14.5 [1.4 – 144.2] | 0.023 | 11.9 [0.6 – 231.6] | 0.101 |
| Marijuana use last 3 months | Never used marijuana in life time | 3 [1.5] | 197 [98.5] | Ref | Ref | Ref | Ref |
|  | No | 2 [9.5] | 19 [90.4] | 6.9 [1.0 – 43.9] | 0.041 | 5.0 [0.5 – 42.9] | 0.141 |
|  | Yes | 2 [3.8] | 50 [96.1] | 2.6 [0.4 – 16.1] | 0.297 | 0.3 [0.0 – 4.6] | 0.402 |
| Tobacco use last 3 months | Never used tobacco in life time | 4 [1.7] | 220 [98.2] | Ref | Ref | Ref | Ref |
|  | No | 0 [0.0] | 25 [100.0] | Empty | - | - | - |
|  | Yes | 3 [12.5] | 21 [87.5] | 7.8 [1.6 – 37.4] | 0.010 | 5.8 [0.5 – 66.5] | 0.152 |

*PEP – Post exposure prophylaxis

**PreP – Pre-exposure prophylaxis
